# Response to letter to the editor

**DOI:** 10.1007/s00259-022-05900-y

**Published:** 2022-07-09

**Authors:** David Kersting, Miriam Sraieb, Wolfgang Peter Fendler, Florian Büther, Christoph Rischpler

**Affiliations:** 1 Department of Nuclear Medicine and German Cancer Consortium (DKTK), University Hospital Essen, University of Duisburg-Essen, Hufelandstrasse 55, 45147 Essen, Germany; 2 Department of Nuclear Medicine, University Hospital Muenster, University of Muenster, Muenster, Germany

Dear Sir,

We thank Dr. Zogala and colleagues for their interest [1] in our recent work on clinical interpretation of [68 Ga]Ga-DOTA PET images and glomerular filtration rate (GFR) estimation by compartmental kinetic modelling [2]. Here, we respond to the specific comments and observations they have made.

Lack of an accurate GFR reference standard to compare to the PET-derived GFR values is a limitation of our study as we already addressed in our manuscript. As current GFR values determined with other methods were not available for the data set we analysed, we decided to compare PET-derived GFR values to serum creatinine-derived GFR values. Dr. Zogala and colleagues correctly state, as we also discussed in our manuscript, that serum creatinine-derived measurements cannot be regarded as absolute gold standard for GFR estimations. Nevertheless, it is a clinically well established and validated metric and a gold standard to assess kidney function in clinical routine [3]. Moreover, the determination of a gold standard GFR at a timepoint close to the PET examination would require additional clinical examinations which was not covered by our study protocol. In future prospective studies, the GFR might be derived from the inulin clearance; the procedure is radiation-free but not widely available [4]. Alternatively, nuclear medicine examination methods might be used, but would demand multiple tracer injections (PET and scintigraphy tracer) within a short time interval [5]. In our study, we transfer a methodology (compartmental kinetic modelling) which was previously described by Lee and colleagues [6] for preclinical PET investigations to a human data set. Nevertheless, we encourage future prospective investigations and are open to share our experiences.

Since the creatinine-derived GFR is not an accurate measure, statistical analysis in terms of accuracy and relative error in comparison to a gold standard was not performed in our study. For the comparison of two methods that are subject to error, correlation analysis and Bland–Altman analysis are established statistical methods that have already been used in many publications including various studies on PET-derived GFR measurements [6–8] and were, therefore, also selected for our work.

A highly interesting suggestion by Dr. Zogala and colleagues is to analyse the dynamic PET data sets using a Patlak model to estimate the GFR. The Patlak analysis is an alternative mathematical approach to kinetic modelling of the full time-activity curves that we performed and is based on a simplified graphical solution to solve the differential equation system. It was established to calculate the GFR from MR or CT data [9, 10] and can, in principle, also be applied to dynamic PET data. However, with only one published study, to the best of our knowledge, within recent years dealing with PET-derived GFR using Patlak analysis [8], it is not yet well-established for this data type. For planar scintigraphy, the Patlak method is used to calculate side separation of renal function; however, as far as we are aware, a direct GFR calculation from planar scintigraphy images is not possible. Significant factors that make the application of a Patlak analysis of dynamic PET data challenging include the lower resolution and greater image noise in comparison to CT or MR data. Therefore, a strong smoothing of the PET-derived time-activity curves is necessary before analysis. In addition, there are further complicating factors like the unclear handling of the temporal window of Patlak analysis after bolus injection. Although a correlation with the reference GFR was shown in a previous evaluation of FDG PET/MRI data [8], due to these specific problems, the Patlak analysis, in our view, is not the method of choice for dynamic PET data and kinetic modelling of the complete time-activity curves in a 1-tissue compartment model appeared to be more promising. Moreover, first attempts of full kinetic modelling for PET data had previously been described [11]. Therefore, the aim of our work was to systematically evaluate a method that has recently also been described for preclinical data [6] on clinical human PET data sets. We plan to look more closely at the application of the Patlak method for GFR calculation from dynamic PET data in future projects. Even if it will most likely also be prone to errors, a comparison of the two methods might allow further insight into the peculiarities and pitfalls of GFR estimations from renal PET data.

With regard to the further points on that Zogala and colleagues comment in their letter, we agree that in the Introduction section it could have been stated more clearly that [^51^Cr]Cr-EDTA is not established for imaging. Additionally, in our evaluation, we compared all GFR values in units of [ml/min]. We confirm that GFR values derived from the CKD-EPI equation were first divided by 1.73 and then multiplied with the body surface area for conversion to comparable units. Concerning Figures 6 and 7 in the original publication, we now provide numerical axis labels in the attached Figures 1 (corresponding to original Figure 6) and 2 (corresponding to original Figure 7).

**Fig. 1:**
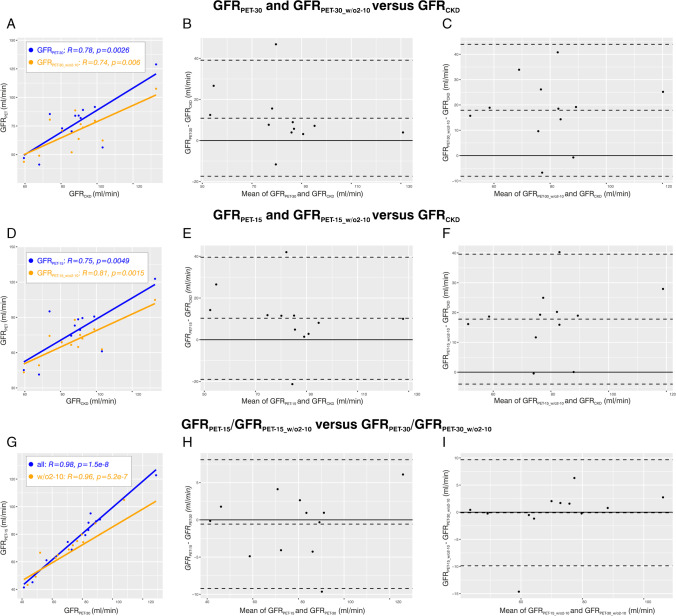
Correlation and agreement analyses for all patients (*n* = 12). **A** Scatter plot for GFR_PET-30_ and GFR_PET-30_w/o2to10_ versus GFR_CKD_. **B** Bland–Altman plot for GFR_PET-30_ versus GFR_CKD_. **C** Bland–Altman plot for GFR_PET-30_w/o2to10_ versus GFR_CKD_. **D** Scatter plot for GFR_PET-15_ and GFR_PET-15_w/o2to10_ versus GFR_CKD_. **E** Bland–Altman plot for GFR_PET-15_ versus GFR_CKD_. **F** Bland–Altman plot for GFR_PET-15_w/o2to10_ versus GFR_CKD_. **G** Scatter plot for GFR_PET-15_ versus GFR_PET-30_ and GFR_PET-15_w/o2to10_ versus GFR_PET-30_w/o2to10_. **H** Bland–Altman plot for GFR_PET-15_ versus GFR_PET-30_. **I** Bland–Altman plot for GFR_PET-15_w/o2to10_ versus GFR_PET-30_w/o2to10_

**Fig. 2:**
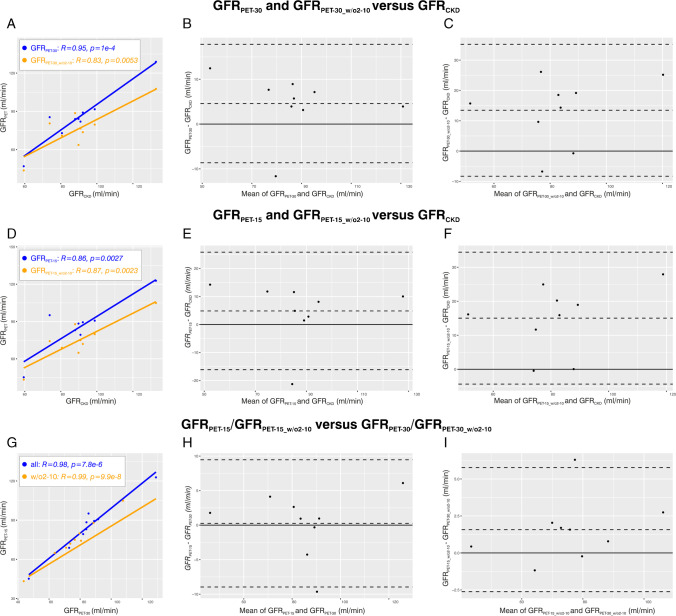
Correlation and agreement analyses for patients with undisturbed urinary efflux (*n* = 9). **A** Scatter plot for GFR_PET-30_ and GFR_PET-30_w/o2to10_ versus GFR_CKD_. **B** Bland–Altman plot for GFR_PET-30_ versus GFR_CKD_. **C** Bland–Altman plot for GFR_PET-30_w/o2to10_ versus GFR_CKD_. **D** Scatter plot for GFR_PET-15_ and GFR_PET-15_w/o2to10_ versus GFR_CKD_. **E** Bland–Altman plot for GFR_PET-15_ versus GFR_CKD_. **F** Bland–Altman plot for GFR_PET-15_w/o2to10_ versus GFR_CKD_. **G** Scatter plot for GFR_PET-15_ versus GFR_PET-30_ and GFR_PET-15_w/o2to10_ versus GFR_PET-30_w/o2to10_. **H** Bland–Altman plot for GFR_PET-15_ versus GFR_PET-30_. **I** Bland–Altman plot for GFR_PET-15_w/o2to10_ versus GFR_PET-30_w/o2to10_

## Data Availability

N/A
